# Successful penile reimplantation after 8 h post penile self‐mutilation: A case report

**DOI:** 10.1002/ccr3.7565

**Published:** 2023-06-13

**Authors:** Mohammed Saleh E. Khalifa Salem, Abdul Alherek, Francis Muangalayi, Alain Kabongo Tshiala, Alain Mwamba Mukendi

**Affiliations:** ^1^ Division of Urology, Department of Surgery, Klerksdorp Hospital University of the Witwatersrand Klerksdorp South Africa; ^2^ Department of Urology Chris Hani Baragwanath Academic Hospital Johannesburg South Africa

**Keywords:** depression, multidisciplinary approach, penile self‐mutilation, psychiatric disorders, reimplantation

## Abstract

**Key Clinical Message:**

Penile self‐mutilation may result from a suicidal attempt during a major depression crisis. The management of this urological emergency should be multidisciplinary. A macroscopic penile reimplantation performed meticulously by a urological surgeon may yield an excellent cosmetic and functional outcome.

**Abstract:**

Penile self‐mutilation is an infrequent form of self‐harming behavior seen primarily in patients with schizophrenia spectrum disorders and rarely reported in those with major depressive disorders.

We herewith present a major depression related case of penile self‐mutilation successfully managed by macroscopic penile reimplantation performed 8 h after the incident.

## 1 INTRODUCTION

Penile self–mutilation (PSM) is a rare urological emergency often associated with psychiatric disorders. Penile mutilation in general may result in life‐threatening hemorrhage therefore it requires immediate surgical intervention.^1^, ^2^, ^3^

The first PSM mentioned in the English literature was by Strock in 1901.^4^ Many cases have been reported in African countries like Kenya and Nigeria.^5^ Self‐inflicted injuries to external genitalia range from small cuts and lacerations to more complex injuries like penile amputation which is a serious challenge to the urologist.

The gold standard treatment for penile amputation is early reimplantation which if successful should yield satisfactory functional and cosmetic results.^1^, ^2^, ^3^

## 2 CASE REPORT

A 33‐year‐old South African man and prison inmate was rushed to the accident and emergency unit of Klerksdorp/Tshepong hospital complex following a self‐inflicted penile amputation and neck laceration due to depression using a sharp knife. He was referred from a correctional service center in Northwest province to the Tshepong facility that deals with trauma emergency cases and presented about 2 h following injury. Further interrogation revealed that he is known to have a depressive illness. There was no history of drug or alcohol abuse.

**FIGURE 1:**
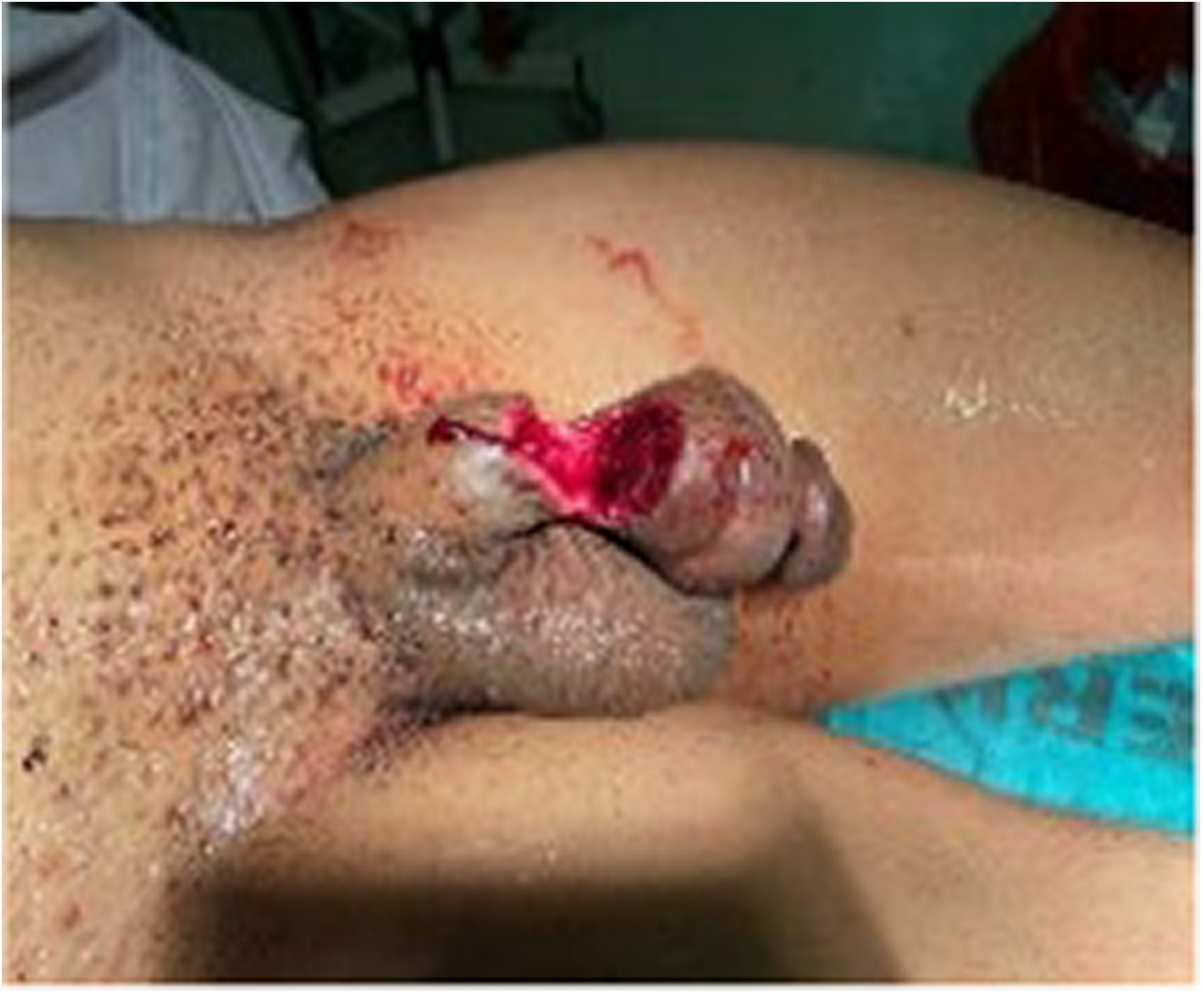
Preoperative image showing almost completely severed penis at midshaft dorsally.

On examination, he was hemodynamically stable, fully conscious, alert but in pain, depressed, anxious, and had a wound on his left side in the middle zone of the neck and an almost completely severed actively bleeding penis (Figure 1). Bleeding was controlled by applying pressure to the wound with gauze swabs. The patient was initially managed according to Advanced Trauma Life Support principles and tetanus prophylaxis was given. The results of hemoglobin, serum urea, electrolytes, and creatinine were within normal limits. A computed tomography (CT) angiogram scan was done and showed no major vascular injury in the neck. Due to slate congestion to access CT angiogram, about 5 h had passed from just waiting for images before being transferred to the Klerksdorp part of the complex to the urology team that took over the management of the patient and took him immediately to theater for an emergency surgery. The initial plan was to excise the distal portion of the penis and proceed to completion of partial penectomy. The patient was also counseled about a possible attempt to macroscopic reimplantation. Upon intraoperative reassessment revealing that the distal penile shaft was still viable. The decision was made to rather go for reimplant with the hope to achieve good cosmetic and functional outcomes.

**FIGURE 2:**
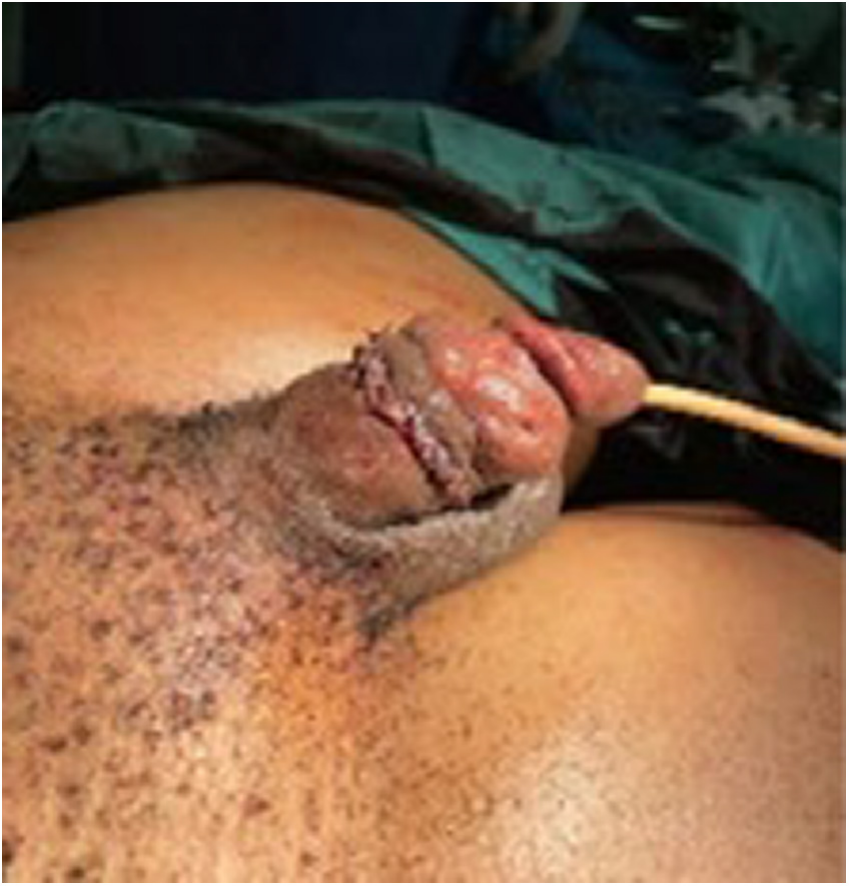
Immediate postsurgery image showing successful anastomosis and catheter in situ.

The urethra was refreshened, spatulated, and reanastomosed over a 16 French catheter in a tension free fashion using continuous 4/0 vicryl sutures. Both cavernosal bodies were sutured using interrupted 2/0 vicryl sutures, the neurovascular bundles approximated with interrupted 4/0 vicryl sutures and the skin closed with chromic 3/0 (Figure 2). The surgery lasted about two and a half hours.

On day 1 postoperative, the patient was seen by the psychiatrist who concluded that he had major depression. The patient was kept in the ward for a week then discharged on antibiotics, analgesia, and a transurethral catheter.

Postoperative course was grossly uneventful, a very small area of skin necrosis was noted on the distal part of the glans in the first week which was almost completely healed altogether with the wound by 4 weeks when reviewed as an outpatient and the transurethral catheter was removed.

**FIGURE 3:**
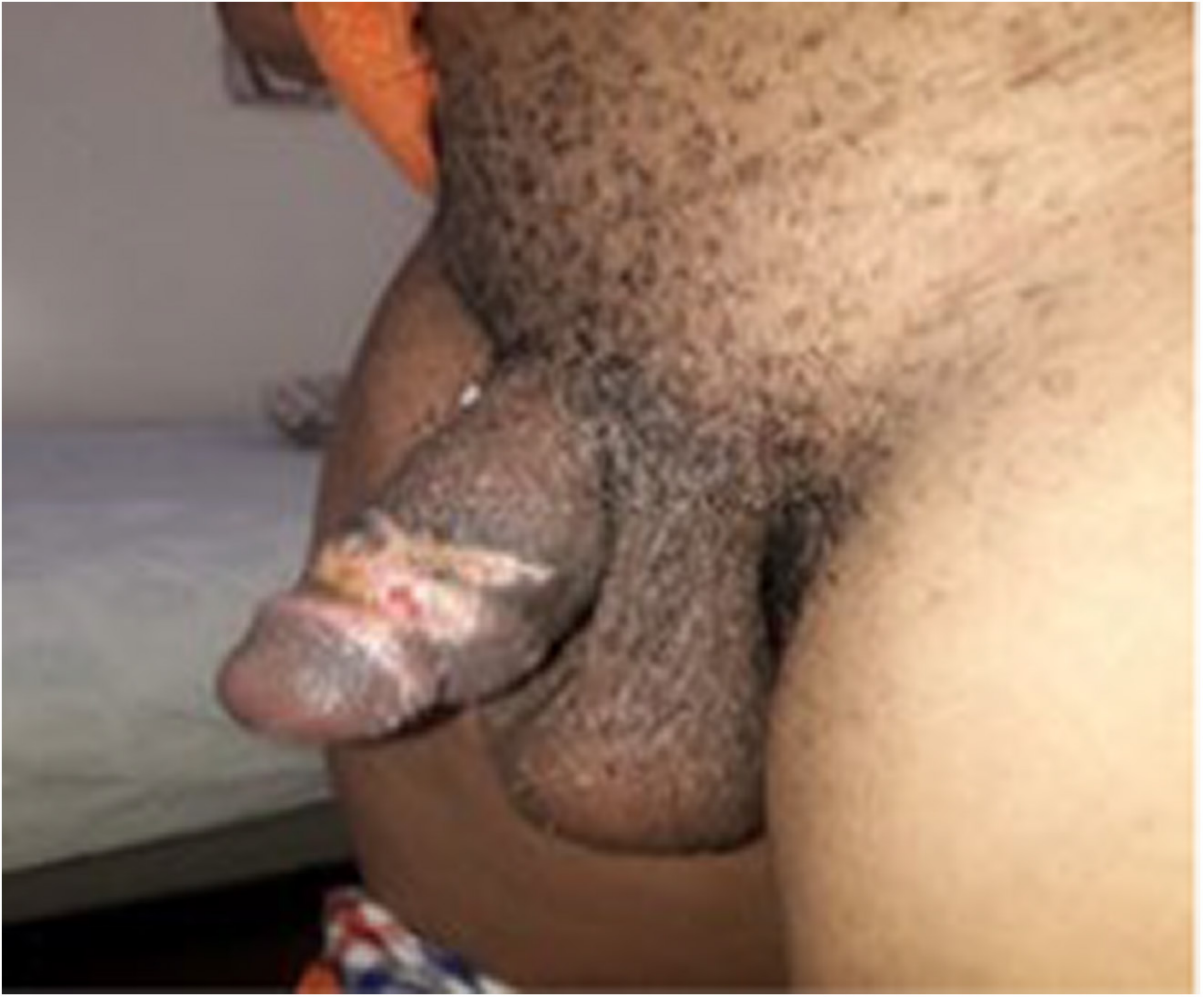
Eight weeks postsurgery image showing good healing in process.

At 8 weeks postoperative, he reported good and rigid erection with normal ejaculation but no sensation on the glans and up to 3 cm proximally. The wound showed good cicatrization in progress (Figure 3) and we noticed a mild meatal stenosis for which a dilatation was done. The distal penile shaft felt more firmer than the rest of the penis. There was no blood flow within the cavernosal bodies in the distal part of the phallus on color Doppler ultrasound.

**FIGURE 4:**
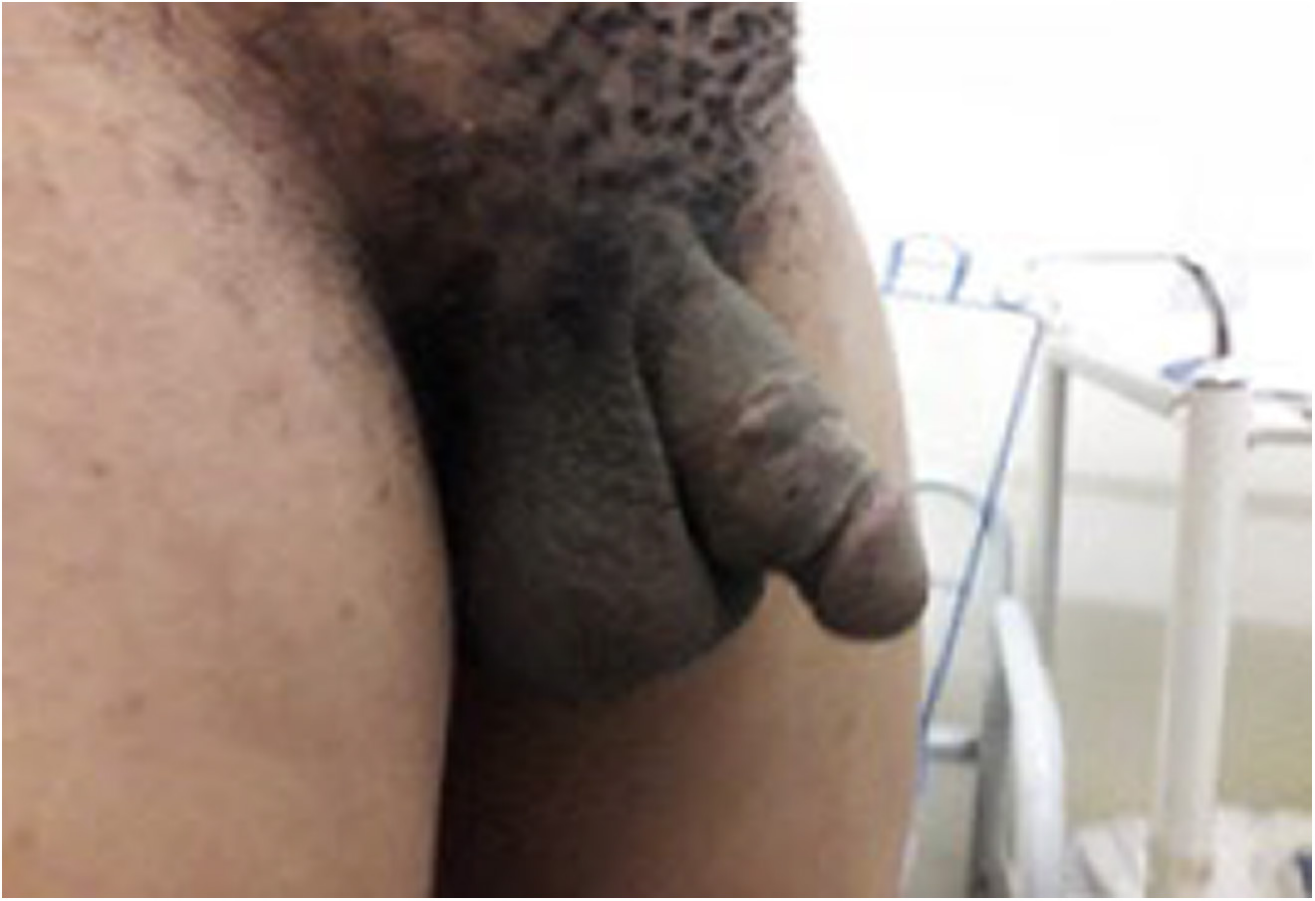
Four months postsurgery image showing good cosmetic result.

At 4 months follow‐up visit, he reconfirmed having good erections during masturbations and reported in addition, a full recovery of sensation on the penile skin. However, the glans was still numb. The phallus looked cosmetically satisfactory (Figure 4) with a meatal stenosis which was dilated, and he was advised to do intermittent self‐dilation.

## 3 DISCUSSION

PSM may be a rare phenomenon often associated with psychiatric disorders such as schizophrenia spectrum; substance abuse; personality and gender dysmorphic disorders. Bipolar and depression disorders are very uncommon associated condition with very few cases reported.^1^, ^3^ The present case was associated with depression, and there was no substance abuse or psychosis.

There appears to be an increasing incidence but whether this is due to an increased level of reporting in recent years remains unclear.^1^, ^3^ Although the act was considered unknown in Africa.^6^ The overwhelming majority of reported incidents occurred among single, Caucasian males in their 20s and 30s.^7^ Our patient fell within the age categories apart from being of African descent.

The primary major literature review on PSM was published in 1979 with few cases of genital self‐mutilation reported thereafter involving both genders.^3^, ^4^, ^8^ The degree of mutilation, the predisposing factors, and therefore the instruments used in the perpetration of this irrational and dastardly act varies.^5^ The instruments may include knives as utilized by the patient in this case; blades; scissors; chainsaw, and axe. Injuries sustained range from simple lacerations of the scrotal skin to complete penile amputation. Our case had sustained an almost completely severed penile injury.

The motivational factor liable for PSM varies. About one‐tenth of self‐mutilators intended suicide secondary to depression and anxiety.^9^ PSM has been categorized either into 3 diagnostic subgroups: schizophrenic patients, transvestites, and people with complex religious and cultural beliefs or into phallicide without psychosis and klingsor syndrome with psychosis.^3^, ^10^ In ours, phallicide is more appropriate.

There is a need for close follow‐up of depressed patients to detect any overt psychotic tendency in the future. It is suggested that in the presence of overt psychotic symptoms, PSM should be considered related to psychotic depression.^11^, ^12^ As part of multidisciplinary team management, psychiatric team assessed our patient and confirmed that he was not psychotic. Early psychiatric involvement was crucial to ascertain that there is no risk of repeating self‐mutilation of replanted penis.

The amputated phallus should be wrapped in saline‐soaked gauzes and placed in a sealed plastic bag which is stored in ice slush “bag in bag”.^2^ Our patient had his amputated penis still attached ventrally to a stalk but covered with saline moistened gauzes. He spent about 7 h before from the incident to the time he was seen by the urology team and taken immediately for emergency theater.

Early replantation of the amputated penis is the gold standard. There have been reports of successful microscopic and macroscopic reimplantation. Distal penile amputation is technically difficult to repair, particularly vascular anastomosis due to small vessels.^2^, ^8^

The amputation in this case was at mid penile shaft and no microvascular technique was used.

Ischemic time is paramount for a successful reimplantation. Tissues with less muscles such as penis can survive 24 h of ischemia. Successful reimplantations have been carried out with a cold ischemic time of fewer than 16 h.^2^, ^3^, ^8^ Whereas some researchers have attempted anastomosis with a cold ischemic time of more than 16 h with a less favorable result.^13^ The event of microvascular technique has improved success with regard to penile reimplantation. Our patient's ischemic time was about 8 h, and no ice was used. The outcome of the surgery was satisfactory.

Complications resulting from PSM varied consistently with the severity of the injury inflicted and the extent of surgical repair undertaken.^14^ Reported postoperative complications include failed reimplant, male erectile dysfunction, urethral stricture, urinary fistula formation, sloughing of the distal urethra, and penile skin. Of note, if the cause of index injury not attended to properly there is a risk of repeat self‐mutilation, death from excessive hemorrhage, or succumb to suicide by completing it.^15^ Numerous reviews and individual case reports have highlighted the inconsistencies that exist in this subgroup of patients and the myriad of motivational factors behind this unique form of deliberate self‐harm.^16^ The identification of these at risk remains as difficult as ever. Even tougher is identifying those patients at risk of repeating this act and those that will go on to complete the suicide intent.^17^

Our patient was followed several weeks after discharge by the multidisciplinary team and besides the meatal stenosis for which he is currently of self‐intermittent dilatation, none other of the above complications were observed.

## 4 CONCLUSION

Successful management of penile self‐mutilation should include a multidisciplinary team in pre‐ and post‐operative period. It should be treated as an emergency without delays. A multidisciplinary postoperative follow ups are crucial part of care and should reassess holistically the patient to prevent repetition of the self‐harm and completion of the suicidal act.

Despite the severity of the trauma involving the penis and taking into account the ischemic time, replantation should be considered as part of therapeutic options guided by pre and intraoperative re‐evaluation. A final and definitive intraoperative decision as in our case led to a successful reimplantation with satisfactory cosmetic and functional results.

## AUTHOR CONTRIBUTIONS

**Mohammed Saleh E.Khalifa Salem:** Writing – original draft. **Abdul Alherek:** Writing – original draft. **Francis Muangalayi:** Writing – original draft. **Alain Kabongo Tshiala:** Writing – original draft. **Alain Mwamba Mukendi:** Methodology; writing – original draft; writing – review and editing.

## FUNDING INFORMATION

None.

## CONFLICT OF INTEREST STATEMENT

The authors declare no conflicts of interest.

## CONSENT

Written informed consent was obtained from the patient for publication of this manuscript and accompanying pictures. A copy of the written consent is available for review by the Editor in Chief of this journal.

## Data Availability

The data that support the findings of this study are available on request from the corresponding author. The data are not publicly available due to privacy or ethical restrictions.
